# Community and health provider perspectives on the quality of family planning and contraceptive services in Kabwe District, Zambia

**DOI:** 10.1080/26410397.2021.1985945

**Published:** 2021-11-08

**Authors:** Theresa Nkole, Adam Silumbwe, Margarate N. Munakampe, Joanna Paula Cordero, Cecilia Milford, Joseph Mumba Zulu, Petrus S. Steyn

**Affiliations:** a UPTAKE Local Principal Investigator, Gynaecologist, Department of Obstetrics and Gynaecology, Levy Mwanawasa Medical University (LMMU), Lusaka, Zambia; b UPTAKE Data Associate, Lecturer/Researcher, Department of Health Policy and Management, School of Public Health, University of Zambia, P.O Box 50110, Lusaka, Zambia. *Correspondence:* adamsilumbwe@gmail.com; c UPTAKE Data Associate, Department of Health Policy and Management, School of Public Health, University of Zambia, P.O Box 50110, Lusaka, Zambia; d UPTAKE Coordinator, Researcher, UNDP-UNFPA-UNICEF-WHO-World Bank Special Program of Research, Development and Research Training in Human Reproduction (HRP), Department of Sexual and Reproductive Health and Research (SRH), World Health Organization, Geneva, Switzerland; e UPTAKE Qualitative Lead, Researcher, MRU (MatCH Research Unit), Department of Obstetrics and Gynecology, Faculty of Health Sciences, University of the Witwatersrand, Durban, South Africa

**Keywords:** community, contraceptives, family planning, health providers, quality, services, Zambia

## Abstract

Quality family planning and contraceptive (FP/C) services result in positive outcomes such as client satisfaction and sustained use of contraceptives. While most assessments of quality in FP/C services are based on measurable reproductive health outcomes, there is limited consideration of the perspectives and experiences of health providers and community members. This study aimed to address this knowledge gap, by exploring health providers’ and community perspectives on the elements of quality FP/C services in Kabwe district, Zambia. Fourteen focus group discussions and 10 in-depth interviews were conducted in October–December 2016, involving community members, key community stakeholders such as religious and political leaders, health committee members and frontline and managerial healthcare providers. Data were analysed using a thematic approach. According to study participants, quality FP/C services would include provision by skilled personnel with positive attitudes towards clients, availability of preferred methods and affordable products. Additional factors included appropriate infrastructure, especially counselling services spaces and adequate consultation time. Participants stressed the need for reduced waiting time and opportunity for self-expression. The efficiency and effectiveness of service delivery factors, such as information dissemination and community engagement, were also considered important elements of quality FP/C. This study underscores the value of considering both community and health provider perspectives in efforts to improve the quality of FP/C services, with the overall aim of increasing client satisfaction and sustained utilisation. However, service delivery processes must also be addressed in addition to providing for community participation, if quality is to be achieved in FP/C services.

## Résumé

Des services de planification familiale et de contraception de qualité ont des effets positifs, comme la satisfaction des clients et l’emploi suivi de contraceptifs. Si la plupart des évaluations de la qualité des services de planification familiale et de contraception sont fondées sur des résultats de santé reproductive mesurables, les perspectives et les expériences des prestataires de services de santé et des membres de la communauté sont peu prises en compte. L’étude visait à combler cette lacune dans les connaissances, en analysant les points de vue des prestataires de services de santé et des communautés sur les éléments de services de planification familiale et de contraception de qualité dans le district de Kabwe, Zambie. Quatorze discussions par groupes d’intérêt et dix entretiens approfondis ont été réalisés d’octobre à décembre 2016, avec la participation de membres de la communauté, d’acteurs communautaires clés comme des chefs religieux et politiques, de membres des comités de santé ainsi que de prestataires de première ligne et de gestionnaires des soins de santé. Les données ont été analysées à l’aide d’une approche thématique. D’après les participants à l’étude, des services de planification familiale et de contraception de qualité devraient inclure des prestations assurées par des personnels qualifiés avec des attitudes positives à l’égard de leurs clients et la disponibilité des méthodes préférées et de produits d’un coût abordable. Les facteurs additionnels incluaient une infrastructure appropriée, spécialement des espaces pour les services de conseil, et des horaires adéquats pour les consultations. Les participants ont souligné la nécessité de réduire le temps d’attente et d’avoir la possibilité de s’exprimer. L’efficacité et l’utilité des facteurs de prestation des services, comme la diffusion des informations et la participation communautaire, ont aussi été qualifiées d’éléments importants. Cette étude montre combien il est judicieux de tenir compte des perspectives aussi bien de la communauté que des prestataires de santé dans les activités pour améliorer la qualité des services de planification familiale et de contraception, avec l’objectif global d’augmenter la satisfaction des clients et de pérenniser l’utilisation. Néanmoins, il est indispensable d’aborder aussi les processus de prestation des services en plus de prévoir la participation communautaire pour parvenir à des services de planification familiale et de contraception de qualité.

## Resumen

Los servicios de planificación familiar y anticoncepción (PF/A) de calidad producen resultados positivos tales como la satisfacción de las usuarias y el uso continuo de anticonceptivos. Aunque la mayoría de las evaluaciones de la calidad de los servicios de PF/A se basan en resultados mensurables de salud reproductiva, consideran de manera limitada las perspectivas y experiencias de profesionales de salud e integrantes de la comunidad. Este estudio procuró abordar esta brecha de conocimiento explorando las perspectivas de profesionales de salud y la comunidad sobre los elementos de los servicios de PF/A de calidad en el distrito de Kabwe, en Zambia. Se realizaron 14 discusiones en grupos focales y 10 entrevistas a profundidad entre octubre y diciembre de 2016, con integrantes de la comunidad, partes interesadas comunitarias clave, tales como líderes religiosos y políticos, integrantes de comités de salud y prestadores de servicios de salud de primera línea y administrativos. Se analizaron los datos utilizando un enfoque temático. Según los participantes del estudio, los servicios de PF/A de calidad deben incluir prestación por personal calificado, con actitudes positivas hacia las usuarias, y disponibilidad de métodos preferidos y productos asequibles. Otros factores mencionados fueron: infraestructura adecuada, especialmente espacios para brindar consejería, y tiempo de consulta adecuado. Los participantes hicieron hincapié en la necesidad de reducir el tiempo de espera y en la posibilidad de automexpresión. La eficiencia y eficacia de los factores relacionados con la prestación de servicios, tales como difusión de información y participación comunitaria, también fueron consideradas como elementos importantes de los servicios de PF/A de calidad. Este estudio subraya el valor de considerar tanto las perspectivas de la comunidad como las de profesionales de salud en los esfuerzos por mejorar la calidad de los servicios de PF/A, con el objetivo general de aumentar la satisfacción de las usuarias y el uso continuo. Sin embargo, para lograr servicios de PF/A de calidad, también es necesario abordar los procesos de prestación de servicios, además de facilitar la participación comunitaria.

## Background

Over the years, family planning and contraceptive (FP/C) services have been recognised as instrumental in global development efforts through the unlocking of educational attainment opportunities and the promotion of gender equality.^1^ The Sustainable Development Goals target three calls on countries to “ensure universal access to sexual and reproductive health care services, including family planning, by 2030”.^2^ However, the high unmet need for FP/C methods and services continues in most low-middle-income countries (LMICs), particularly among marginalised groups.^3^ For example, in 2017 approximately 20 million women globally needed, but were not using, a modern FP/C method.^4^

To achieve economic growth and harness their demographic dividend, LMICs need, in addition to other socio-economic policy measures, to improve the quality of and access to FP/C services and methods.^3^ Quality of care (QoC) has been documented as a key determinant in influencing the uptake of FP/C services.^5,6^ “QoC” was conceptualised at the International Conference of Population and Development in 1994, as a means to assist couples in achieving their fertility intentions through access to improved family planning programmes.^7^ Providing high-quality care increases the utilisation of FP/C services, resulting in improved reproductive health outcomes.^8^ Conversely, evidence suggests that dissatisfaction and disapproval of service provision modalities result in non-use, non-attendance and discontinuation of service use.^9^

While “quality” in healthcare has various definitions and measurements, it is generally defined as the delivery of the service in an appropriate manner to achieve desired outcomes.^10^ According to the Bruce/Jain QoC framework,^11^ quality in family planning service provision consists of six elements: (1) choice of method, (2) information about the different methods, (3) technical competence of service providers, (4) interpersonal relations, (5) continuity of method and (6) follow-up and an appropriate constellation of services.^11^ Most assessments of quality in FP/C are based on measurable reproductive health outcomes, with limited consideration of the perspectives and experiences of health providers and community members.^12–14^ These perceptions of FP/C methods and services may be context-specific due to different influences such as cultural norms and religious practices, but valuable in informing efforts to provide quality FP/C services.^15^

A number of studies in sub-Saharan Africa assessing quality of FP/C services have focused on the comparison between public and private care provision,^16,17^ while others have addressed a single method, particularly the long-acting reversible contraceptive.^18,19^ However, these efforts to assess the quality of FP/C services provision are inadequate as they mostly emphasise the supply side factors while overlooking the contribution of client experiences to shaping FP/C service quality.^5^ In this study, we explore health provider and user/community perspectives of QoC in order to promote patient-centred care, while also acknowledging the bottlenecks and challenges on the supply side. Moreover, we believe that understanding these perceptions contributes to informing the planning and evaluation of participatory interventions seeking to improve QoC in the provision of FP/C services.

This paper is based on a study that was part of the formative phase of a multi-country project conducted in Kenya, South Africa and Zambia (the UPTAKE project).^20^ The UPTAKE project was the formative phase of a complex-designed intervention that set out to increase met needs for FP/C in the three countries. The intervention aimed to facilitate community and healthcare provider participation in the provision of FP/C services. This paper reports on the exploratory qualitative research conducted during the formative phase which aimed to explore the understandings of the QoC of FP/C service provision, from key informants’, healthcare providers’ and community members’ perspectives, in the Kabwe district in Zambia. Specifically, the paper shades light on how community and health provider participatory interventions can be leveraged to address QoC of FP/C services provision. In addition, given that the UPTAKE project was a multi-country study, aspects of QoC differed depending on the health systems development in the study countries. This study allowed us to explore the QoC of FP/C service provision in a Zambian context.

## Methodology

### Study design

The UPTAKE project was planned to be undertaken in two phases, the formative (intervention development) and implementation (refinement and deployment), respectively. In the formative phase, an intervention to increase FP/C met needs through community and healthcare worker collaboration in the provision of FP/C services, within a human rights framework, was developed.^21^ This phase included exploratory qualitative research to understand the country contexts in which FP/C services are provided, and to identify the human rights domains in which to contextualise the intervention.^20^ The formative qualitative research had four objectives that sought to explore: (1) knowledge, attitudes and practices in FP/C services and utilisation, (2) barriers/enablers to FP/C service provision/access, (3) understandings of QoC and (4) community participation activities and practices in FP/C services. Specifically, this paper presents findings based on the third objective focusing on QoC, while other components of the UPTAKE formative phase are published elsewhere.^20,22–24^ The other manuscripts report on different objectives of the formative research and/or describe findings in different country contexts. Each of the papers provides an in-depth analysis on a different portion of the qualitative data gathered during the formative research.

### Study setting

We implemented the study in Kabwe district, the provincial capital of the Central Province, Zambia. In 2014, Central Province reported a high unmet need for FP/C (25.7%) and a contraceptive prevalence of 42.8%.^25^ Kabwe district had a population of approximately 217,843 people of whom 58,381 (26.8%) were women of reproductive age (15–49 years) in 2014.^25^ Thus, we chose Kabwe as the study district because its population characteristics fit the study requirements, that is, it was sufficiently varied, comprising of rural, urban and peri-urban catchment populations. There was also availability of FP/C services and infrastructure, including existing community participatory structures such as the Neighbourhood Health Committees that manage the work of the Community Health Workers. After discussions with the Kabwe district health office, one health centre providing health services to a catchment area with rural, urban and peri-urban communities was chosen as the study site.

### Study population

We conducted 12 focus group discussions (FGDs) with community members, women and men in the reproductive age group 15–49 years, living within the catchment area of the selected health centre. We included community members as both users and potential users of FP/C, as they could provide perspectives on acceptability and uptake beyond those of only clinic attendees. We also held two FGDs with healthcare providers representing both managerial and frontline staff from healthcare facilities within the Kabwe district. We organised community FGDs by age, marital status, sex and whether participants had children or not (Table 1) to encourage ease of discussions. We included male community members in the study groups as men are often left out of discussions on FP/C, even though they are key in FP/C decision-making.^26^ We held individual in-depth interviews (IDIs) with key stakeholders who were involved in providing FP/C services, as well as community leaders from the Kabwe district (Table 2). Community leaders included religious/church leaders, headmen and managers of non-governmental organisations (NGOs) involved in FP/C service provision.

**Table 1.** Focus groups discussants

Focus group discussion categories	Age range	Participants
**Community members**		
Adolescent girls, urban	15–19	10
Adolescent girls, rural	15–19	09
Young women, urban	20–34	08
Young women, rural	20–34	10
Older women, urban	35–49	08
Older women, rural	35–49	09
Young women, unmarried	20–34	10
Young women, married	20–34	10
Women, no-children	18–49	10
Adolescent boys	15–19	10
Young men	20–34	10
Older men	35–49	10
**Healthcare providers**		
Managerial healthcare provider	-	10
Frontline healthcare providers	-	09
**Total participants**	** **	**133**

**Table 2.** List of in-depth interview participants

Categories	Participants
Political leader	1
Neighbourhood health committee	1
NGO	1
Traditional leader	1
District medical office	1
Provincial medical office	2
Secondary school teacher	1
Religious leader - councillor	1
Religious leader - reverend	1
**Total**	**10**

### Sampling/recruitment process

We purposively sampled the study participants. We identified key informants from the community with the help of the healthcare providers at the selected health facility as well as the district health office. This sampling method ensured the representation of key players in FP/C services, including experts and persons with influence in the health sector. Similarly, we recruited the community FGD participants using purposive sampling to ensure appropriate representation in the different categories. Screening and recruitment of community members took place at schools, churches, clinics, homes (door to door visits) and through referral by other participants. We recruited the health provider FGD participants from across the health facilities in the Kabwe district.

### Data collection

We conducted 14 FGDs comprising 8–10 participants between 16 October 2016 to 4 December 2016. A total of 133 participants (19 healthcare providers and 114 community members) participated in the FGDs (Table 1). Eight facilitators comprising four members of the research team and four interviewers with experience in community participatory programmes conducted the FGDs. Each FGD had one facilitator and one notetaker. We matched interviewers/notetakers with participants by gender. We trained the research team on how to explain the study objectives and administer the research instruments, as well as use of digital recorders during data collection. All FGDs were conducted at the study site at a time that was suitable for all participants. We conducted 10 interviews with key stakeholders and community leaders at a time and place convenient to them (Table 2). We carried out the interviews mostly in English and Bemba. We audio-recorded, transcribed verbatim and translated interviews which were necessary. Given that the formative phase of the UPTAKE project had various objectives, we ensured that rich data were collected on all aspects of the project. This was important because each objective played a distinct role in the planning and evaluation of the complex-designed intervention across the three country contexts.

### Data analysis

We used thematic content analysis to analyse the data.^27^ We used NVivo (Version 10, QSR International) to facilitate organisation, coding and analysis of the data. We read the transcripts for identification of codes related to all the formative research objectives, which were grouped into categories and then into themes.^28,29^ We then developed a single master code-list with code definitions used across the three study countries. We generated codes iteratively based on input from the questions in the interview guides and emergent themes. Following the development of the initial draft of the code-list, a subset of transcripts (representing different FGDs/IDIs from each country) were double coded by the local researchers and compared across countries to ensure reliability and validity of coding. There were multiple iterations of the code-list based on double coding and consultations until an agreement was reached. Once the master code-list was developed, the rest of the coding was conducted at the country level. The data analysis approach was predominantly deductive given that the data were clustered around key thematic areas of the Bruce/Jain framework on principle elements of QoC,^11^ whereas we also employed an inductive approach by exploring the context-specific emergent themes on QoC in the data. A detailed description of the data analysis methodology is documented elsewhere.^30^

### Ethical considerations

We obtained ethical approval from the University of Zambia Biomedical Research Ethics Committee (UNZABREC) (Ref: 003-03-15) to conduct the research, and the study was authorised by the Ministry of Community Development, Mother and Child Health (MCDMCH). The World Health Organization (WHO) Research Ethics Committee and the Research Project Review Panel (RP2) (Project ID A65896) also approved the UPTAKE study. Before commencement of the FGDs and IDIs, an information sheet describing the study was administered to all participants and once they agreed, written informed consent to participate in the study was then provided. Participants under the age of 18 years provided written assent and their parents/guardians provided written consent for their participation. If participants were not literate, a witness was required during the consenting process. The participants also gave separate written consent to be recorded.

## Results

The results are presented according to the elements of the Bruce/Jain QoC framework on the provision of FP/C services.^11^ This framework provides a basis for assessing FP/C services quality from the client's perspective, while recognising key service delivery aspects. We report community members’, health providers’ and key stakeholders’ perspectives specifically on four elements of QoC in the Bruce/Jain framework: (1) the choice of method, (2) information about the different methods, (3) technical competence of service providers and (4) interpersonal relations. In addition, community engagement – which is not captured in the Bruce/Jain framework – emerged from the data as a fifth element to consider in understanding QoC in the Zambian context. Views on the quality FP/C services were mostly similar across the participant types. However, views which were specific to a particular category of participants are reported as such.

### Choice of FP/C methods

#### Availability of family planning methods

Availability of preferred FP/C methods was cited as an important feature of QoC. Community members, including females without children, married young and rural adult females made the most reference to this aspect of quality. They indicated that while the healthcare providers encouraged them to plan their families, their preferred methods needed to be available to all categories of users regardless of their status in society. Unavailability of preferred methods was said to compromise the quality of FP/C services. Furthermore, the availability of a wide variety of products was considered as quality by key informants, healthcare providers and community.

“*I think the other thing is the availability of these methods because you can explain to these couples, but if those methods are not going to be available all the time, then that's poor-quality family planning services*.” [Healthcare provider FGD, Managerial, UZHG_H001]

#### The cost of family planning and contraceptive methods

Community members and the managerial level healthcare providers felt that free access to FP/C methods and services contributed to the good quality of services. Community members narrated how it was difficult to sustain the use of FP/C methods if they had to purchase them from local pharmacies, as most of the community members had insufficient financial resources.

“*If it's cost-effective as we said they are not paying anything, they can access free of charge, so if they can access, they are not buying and the drug is potent then we can say it is quality.*” [Healthcare provider FGD, Managerial, UZHG_H010]

“*Okay, we usually have challenges especially when you go there and find that they have run out of stock with the method that you are on. So, you will have to go and buy the drugs and it is challenging if you don't have money especially that our husbands cannot abstain from sex.*” [Female Urban FGD, 35–49 UZFG_UA003]

#### The nature of spaces for family planning and contraceptives services provision

All categories of participants reported that the state of the institution providing FP/C methods/services was another important determinant of quality. The main concerns described were availability of appropriate infrastructure or room/space, to ensure privacy to FP/C clients. Healthcare providers, specifically, reported that the availability of necessary infrastructure was critical for quality services provision. They indicated that limited space sometimes resulted in reduced confidentiality and privacy. Quality FP/C services should have enough privacy so that clients should not be worried about other users or providers.

“*If rooms were built maybe where you are offering family planning there is a specific room where you can even examine if a client has something to say maybe that needs to be said in private you can easily do that.*” [Healthcare provider FGD, Managerial, UZHG_H006]

Both healthcare providers and community members noted that the provision of FP/C services in separate rooms with relevant equipment was essential for the services to be considered as good quality. Some of the equipment mentioned included that required for the collection of vitals such as blood pressure machines and thermometers. Unmarried young adults and adolescents expressed dissatisfaction with having to access FP/C services in the same place as pregnant women attending antenatal care.

“*I would like that when we go to the clinic, the way they have one room for all services, I would love if women had their own room where they get family planning services. That is what I would love, then I can say it's good quality.*” [Female FGD, Urban Young adult, UZFG_UY003]

“*Good quality of care, I think infrastructure still comes in then the staff who have the knowledge and the skills to me would be key and having what to use in terms of resources.*” [Key stakeholder IDI, Health Sector, SRH-NGO, UZI04]

### Information about the different FP/C methods

#### Effective information dissemination during counselling

Key informants from the health sector, NGOs and community (including the traditional leaders, political and religious stakeholders), all pointed out that the provision of adequate FP/C information to clients was essential for quality services. This included the processes through which dissemination or communication of information about available FP/C services was provided at the community level. It was noted that the provision of comprehensive information on the methods and their side effects was vital during counselling.

“*We need to actually to make sure that enough information is actually circulated in our communities. The information about family planning is only available in health facilities.*” [Key stakeholder IDI, Community, UZI_001]

Key informants reported that information dissemination was currently mostly done in health facilities and by healthcare providers. Some participants indicated the media and information, education and communication materials in the health facilities as sources of information. The youth reported getting information from schools and youth groups in the community. Churches and religious leaders also reportedly provided advice on FP/C. However, they agreed that quality FP/C services entailed clients having the correct and up-to-date information to facilitate informed decisions.

“*If the programs are well planned, all can be well because the health officers providing the services can continue; that is, community sensitisation, awareness in the schools such that they can even train more family planning health workers in the community so that they can help deliver this information in the community.*” [Male FGD 35–49 UZMG_ A002]

#### Allowing community members to express themselves and feed back

At the individual level, the key stakeholders from the health sector highlighted the importance of counselling services for informed client decision-making. Community members, particularly the married young adult females, further narrated that they appreciated FP/C services when they were given an opportunity to dialogue with healthcare providers, express themselves and get feedback on FP/C method options.

“*We can't say they are quality because on one part they do not do everything as they should. They give us the methods but they do not give us the chance to express our feelings fully concerning how you are feeling yourself.*” [Female FGD, Married Young Adult, UZFG_MY004]

### Technical competence of service providers

#### Availability of skilled personnel and time dedicated to service provision

Another key determinant of QoC from both provider and community perspectives was the availability of skilled or qualified personnel at the health facilities, who are equipped with the necessary resources to perform their duties. Furthermore, having a sufficient number of skilled personnel at health facilities also meant that the health workers would be able to provide quality services, as opposed to the hasty manner in which services were being provided due to high numbers of clients.

“*The family planning health workers should be many so that for instance when they get five people, they interview them; find out the problem they are having so that they can know the step they are supposed to take. Because when there is only one person, the person will just concentrate on providing the injection and indicating the day the person is supposed to come back to the clinic without knowing you have something to explain to them.*” [Female Urban FGD, UZFG-UY004]

Frontline-level healthcare providers and community members described how dedicating sufficient time to FP/C services provision was important for quality service provision. Community members felt that in some instances the scheduled time for FP/C services was insufficient, and therefore the providers did not have enough time to engage with clients. In some situations, for example, healthcare providers noted that the days or hours dedicated to FP/C services were insufficient. Furthermore, quality FP/C services were also said to be those with less waiting times.

“*If we had enough room, it is possible that you can have from morning up to 16:00 hours doing family planning. Now because we have challenges with space you find that that’s why family planning is limited for two hours which is from 14:00 to 16:00 hours.*” [Frontline provider, UZFG_H008]

#### Limited capacity to offer long-acting methods

Managerial health providers and key informants all agreed that there were providers at some of the health facilities who were not trained to offer certain FP/C methods, particularly the long-acting ones. The lack of training compromised quality as it entailed that such providers could not confidently offer certain methods to the clients. However, some key health sector stakeholders reported that there were efforts to build capacity to offer long-acting methods among providers through a training programme spearheaded by the government and the cooperating partners in the district.

“*The type of method availability can be considered quality. For example, if a woman wanted to have Jadelle [contraceptive implant] for five years and then you say no we don’t have that method. Automatically it has compromised the quality. And then the information also from the provider, if the provider has not much information as well as the capacity to give such a method to the client. It compromises the quality.*” [Healthcare provider FGD, Managerial, UZHG_H007]

### Interpersonal relations

#### Healthcare provider conduct/attitudes

Though both healthcare providers and community members considered positive healthcare provider attitudes and good conduct as key to QoC FP/C service provision, the community members made the most reference to this. Positive attitudes and good conduct included being shown respect by the health workers, having attention paid to their needs, and being allowed to express themselves freely with regard to the FP/C services/methods they wanted. The community members narrated that it was important for healthcare providers to be able to understand them and offer the required services, especially when they had concerns about side effects. It was also reported that in some cases clients, especially the young and unmarried, avoided the FP/C services because healthcare providers asked them judgmental questions which clients perceived as discriminatory, based on their marital status. While marital status increased accessibility, some young married women were still treated with judgement for accessing services:

*“Youths in most cases when they go to the health facility to access the family planning the attitude of caregivers, they send them away when they are scolded to say you're still in school you're supposed to concentrate on books and you're coming for family planning at your age, you're going to fail, you're going to get sick. They normally shun from going ahead with their intentions. So, if we stigmatise that child or that youth, they keep away from the health facility and as a result, they fall victim to early pregnancies and other STI's.”* [Healthcare Providers, Managerial FGD, UZHG_H004]

*“We are asking that when we go to the clinic for family planning, they should understand what a client says one on one because others just come and say I want the one for three months and when they go back to complain about the side effects should be treated according to their explanation. Sometimes they embarrass you in the presence of others. They should at least listen to the clients and their concerns and attend to them accordingly. That is the provision of quality FP/C services.”* [Female Married Young Adult, UZFG_MY004]

### Community engagement in FP/C services

Community members indicated that quality FP/C services needed to accommodate community perspectives. They highlighted the need for platforms where they can participate in the planning and delivery of family planning and contraceptive services. Furthermore, they indicated that quality FP/C services needed to use people from the target communities to provide such services as FP/C information. The health providers narrated that engaging the community would help them understand the quality of FP/C services provided; compliments from the community would indicate quality services, while dissatisfaction would manifest in negative talk.

“*If they can get people from our communities and educate them to reach out within our own communities. As a Bemba saying goes, “akachila kambushi kasengula apo kekele” [one must first take care of the place where one lives before going to do the same elsewhere.] They should pick from the areas where we stay and educate them. These will be our eyes, our ambassadors, they know our problems. Those will know that at a particular time, they can be going to the district to get the medicine to talk to the people.”* [Females rural adult, 35–49, UZFG_RA001]

*“Just to add on what my colleague has mentioned also, I think it’s also were you, were, you, you the attitude of the staff is up there, eh. Compliments from the community itself also giving more information about the quality of services being offered, if they keep on praising the services that you are giving to them according to the health, ah community perspective then you are offering quality services.*” [Frontline Provider, UZFG_H007]

## Discussion

Several issues were considered as key in defining QoC in FP/C services from both the community and the healthcare providers’ perspectives. These included the availability of methods and infrastructure, provision of adequate FP/C information, having skilled FP/C services healthcare providers, positive healthcare provider attitudes such as treating clients with respect and understanding their specific needs. The engagement of community members in FP/C services was also highlighted as a key component of quality services. These findings coincide with major frameworks on ensuring quality in FP/C services.^11,21^ They underline the value of considering the perspectives of both health providers and community members in understanding QoC in FP/C services. In addition, our findings contribute to understanding how community and health provider participatory interventions can be leveraged to address aspects of QoC in FP/C services provision at lower levels of the health system.

The availability, as well as the cost of certain FP/Cs, were important considerations in the choice of method. Other studies have found, equally, that a wider method mix, readily available and less costly options in cases where public facilities have run out of FP/C methods provide an opportunity to satisfy women’s FP/C needs.^31,32^ Community members and health providers agreed on the significance of infrastructure that guaranteed the privacy of clients as vital in ensuring quality FP/C services. Apart from being a deterrent to access, visual and auditory privacy influence the choice of contraceptive methods.^5^ Limited privacy could result in reduced use of a particular method, for fear of being identified by others. Clients may opt for methods associated with less risk of breach of confidentiality and privacy.^33^ Breach of privacy was said to occur during discussions on issues of sexuality, as well as the provision of methods like Jadelle (implant). However, the participants did not refer to amenities such as water, toilets and availability of electricity as important determinants of choice.

Strategic communication and information dissemination to ensure client needs are met was another critical element of quality FP/C services. Information dissemination on available contraceptive methods, their advantages and side effects were cited as key to improving the quality in FP/C service provision. Information dissemination improved informed decision-making and dispelled some of the myths and misconceptions associated with FP/C. Providing more comprehensive and accurate information that is tailored to the client’s needs has been associated with higher client satisfaction, client retention and a higher contraceptive prevalence rate.^34^ Access to information, especially in the rural areas through outreach activities, was noted as a way to increase FP/C service utilisation.^35^ However, the FP/C service providers were also encouraged to use innovative and context-appropriate approaches, not only to create awareness, but also to empower couples’ reproductive decision-making.

The attitude of providers towards clients was considered an important element of QoC. Negative provider attitudes discourage community members from discussing their FP/C needs and hinder certain groups, such as the unmarried and young people, from accessing FP/C services. Interpersonal relations were more of a concern to the community members than the healthcare providers. The provider's tone, manner and modes of speech were important to clients.^36^ In One of the study in the Democratic Republic of Congo, where women were asked which qualities were best for a nurse, they indicated: communication style, respect, attentiveness and technical qualities.^37^ The client–provider interaction was important for the initial adoption of a method, effective usage and continuation, as well as “word of mouth” publicising of reproductive health services within the community.^38^

Both the healthcare providers and the community members recognised the role that community plays in shaping the quality of services provided. This finding suggests the importance of moving beyond formal health systems factors such as professional conduct, health products and institutional factors to include community-based health systems such as community engagement in disseminating FP/C information in efforts to improve the quality of health services. Community participation has been underscored as one of the key principles of providing FP/C services within a human rights framework.^21^ Involving the community not only ensures utilisation but also facilitates accountability in FP/C services provision.^23^ However, facilitating participation in FP/C services will require building trust in FP/C methods/services: for example, promoting facilitative strategies that address structural failures such as the feminisation of FP/C services.^23^

Apart from improving QoC, the attributes of quality FP/C services, as identified by the community and healthcare providers, were important because of their potential to contribute towards overall improved health services coverage and utilisation. While some of the issues brought out may require longer-term response strategies, interventions that seek to improve quality in health services process may prove vital.^39^ This may include training to enhance capacity to provide advanced FP/C methods as well as improving local commodities supply systems.^37,40^ Interventions to improve QoC must also address limitations at the organisational and community levels as efforts to overcome a particular constraint are less likely to be successful if this interdependence is not taken into account.^23^ Governments should continue to implement and encourage incentives, perhaps performance-based, as well as strong regulatory and monitoring structures to ensure quality FP/C services.^41^ Efforts to improve quality of FP/C services should also incorporate components of person-centred quality care such as dignity, social support and supportive care to have the highest likelihood for success.^42^

QoC is one of the dimensions of human rights that needs to be systematically and comprehensively considered in the rights-based provision of sexual and reproductive health services.^21^ This is because QoC is overarching and addresses various human rights aspects such as non-discrimination, availability of services, accessibility of services, informed decision-making, privacy and confidentiality, participation, respect, protection and fulfilment of all individuals using or wanting to use FP/C services. Further, QoC is one of the measurable and comparable human right domains for participatory programmes aiming to meet unmet needs for FP/C low-resource settings such as Zambia.^20^ Therefore, the findings of this study are valuable in the efforts to ground sexual and reproductive health programmes, including FP/C services, within the human rights framework, in order to improve access to services.

Our findings further highlight aspects of QoC that can potentially be addressed through the community health system, which is defined as a set of local actors, relationships and processes engaged in producing, advocating for and supporting health in communities, but existing in relationship to established health structures.^43^ The collective use of community resources/actors to provide better QoC of FP/C services for all has been termed as reactivating the community health systems.^44^ We acknowledge that while certain aspects of QoC go beyond the capacity of community health systems, community actors with linkages to the formal health system, such as neighbourhood health committees, community health workers, public health nurses and community-based distributors, not only play an essential role in providing FP/C services but also in advocating for QoC at the health facility level.

### Strengths and limitations of the study

To enhance the credibility and dependability of findings, we systematically and comprehensively reviewed all the collected qualitative data.^45^ The team, comprised of experienced qualitative researchers within the UPTAKE project from Zambia, Kenya, South Africa and the WHO, oversaw the process of data analysis including providing input in the development of the master code-list containing key country-specific thematic areas. The use of the Bruce/Jain framework may have masked some potentially cross-cutting data across country teams. However, this cross-country data analysis process ensured transparency and sense-making by all team members.^46^

The study was exploratory in nature and was conducted in one setting with a single purposive sample of respondents in the Kabwe district. However, the findings may be transferable to other settings with similarities to the catchment population of the study health facility. Further, the study counted on various categories of providers, both current and potential FP/C services users within the reproductive age range, which not only enabled triangulation of views but also a detailed exploration of experiences with FP/C services across different categories. We also acknowledge that, being exploratory, this study may not have been able to delve deeply into some of the participant category specifics with regard to FP/C services used in the study setting. Therefore, these findings could be used in addition to other studies to uncover more in-depth information on FP/C services with further analysis of some of the individual participant categories used in this study.

## Conclusion

The community and health providers indicated the necessity of quality FP/C services that included: appropriate facility infrastructure, skilled healthcare providers, positive healthcare provider attitudes such as treating clients with respect and understanding their needs, the provision of clients’ preferred methods with adequate information and engagement of community members in FP/C services. In exploring the appropriate standards of quality, this study underscores the value of considering both community and health provider perspectives in efforts to improve the quality of FP/C services, ultimately increasing utilisation. It is worth noting that improving the quality of FP/C services plays a major role in addressing high unmet needs, as well as increasing satisfaction and continuity. However, efforts to improve quality must address service delivery challenges and facilitate community participation if community perspectives are to be considered in improving the QoC of FP/C services. Future research must, therefore, seek to explore how to integrate community perspectives and healthcare provider views, in measuring the quality, as well as use of these perspectives in designing person-centred quality FP/C care programmes for the communities.

## Data Availability

The data are not publicly available as it contains information that could compromise research participant privacy/consent. However, some anonymized aspects of the datasets may be available upon request and with permission of the Department of Sexual and Reproductive Health and Research, World Health Organization. Note that data sharing is subject to WHO data sharing policies and data use agreements with the participating research centres.
